# Febuxostat attenuates secondary brain injury caused by cerebral hemorrhage through inhibiting inflammatory pathways

**DOI:** 10.22038/IJBMS.2024.74655.16212

**Published:** 2024

**Authors:** Yang Bai, Hongxia Shi, Ying Zhang, Chenyu Zhang, Bin Wu, Xinghan Wu, Zhenwei Fang, Qi Wang, Xiutian Sima, Tiejun Zhang

**Affiliations:** 1 Department of Pharmacy, West China Hospital, Sichuan University, Chengdu, Sichuan, China; 2 West China School of Pharmacy, Sichuan University, Chengdu, Sichuan, China; 3 Department of Neurosurgery, West China Hospital, Sichuan University, Chengdu, Sichuan, China; # These authors contributed equally to this work

**Keywords:** Bioinformatics analysis, Febuxostat, Inflammation, Intracerebral hemorrhage, NLRP3 inflammasome, Second brain injury, Trend analysis

## Abstract

**Objective(s):**

Neuroinflammation is considered an important step in the progression of secondary brain injury (SBI) induced by cerebral hemorrhage (ICH). The nucleotide-binding and oligomerization structural domain-like receptor family of pyridine structural domain-containing 3 (NLRP3) inflammasomes play an important role in the immune pathophysiology of SBI. Febuxostat (Feb) is a xanthine oxidase inhibitor that is approved for the treatment of gout and has been found to have potent anti-inflammatory effects. However, it has been less studied after ICH and we aimed to explore its protective role in ICH.

**Materials and Methods:**

We established an autologous blood-brain hemorrhage model in C57BL/6 mice. Functions of co-expressed genes were analyzed by trend analysis and bioinformatics analysis. Enzyme-linked immunosorbent assay were used to assess the inflammatory factor levels. Fluoro-Jade B histochemistry and TUNEL staining were used to detect neuronal apoptosis. Immunofluorescence staining and western blotting were used to detect the expression of NLRP3 inflammasomes.

**Results:**

Pretreatment with Feb reduced neuronal cell death and degeneration and alleviated neurobehavioral disorders *in vivo*. Feb was found to modulate inflammation-related pathways by trend analysis and bioinformatics analysis. In addition, Feb inhibited microglia activation and elevated cytokine levels after ICH. Furthermore, double immunofluorescence staining showed that co-localization of NLRP3 with Iba1 positive cells was reduced after treatment with Feb. Finally, we found that Feb inhibited the activation of the NLRP3/ASC/caspase-1 pathway after ICH.

**Conclusion:**

By inhibiting the NLRP3 inflammasome, preconditioning Feb attenuates inflammatory injury after ICH. Our findings may provide new insights into the role of Feb in neuroprotection.

## Introduction

Intracerebral hemorrhage (ICH) is the second most common subtype of stroke and a serious public health problem with high rates of death and disability (1, 2). Primary ICH damage to the brain is due to the early formation of a hematoma following hemorrhage, resulting in mechanical damage (3, 4). Accumulating evidence has demonstrated that microglia are the major cell type responsible for secondary brain injury (SBI) after ICH owing to the release of cytokines, chemokines, and other immunoreactive molecules (5, 6), as well as the presence of oxidative and inflammatory stresses (7). 

The nucleotide-binding and oligomerization domain-like receptor family pyrin domain-containing 3 (NLRP3) inflammasome is a critical component of the innate immune system (8). It mainly consists of a cytosolic sensor molecule NLRP3, an apoptosis-associated speck-like protein containing a caspase activating recruitment domain (ASC), and procaspase-1 (9, 10). The over-activation of NLRP3 inflammasome is a process of inflammatory responses that has been implicated in diverse neurological diseases, such as cerebral ischemia (11), intracerebral hemorrhage (12), migraine (13), and brain infection (14). The NLRP3 inflammasome can be found in multiple brain cell types, particularly in microglia cells, acting as the first immune defense in the central nervous system (15). Increasing studies indicate that the activated NLRP3 inflammation in microglia is a potent driver for neuroinflammation (13, 15, 16).

Febuxostat (Feb) is a xanthine oxidoreductase inhibitor that has been developed to treat chronic gout. Recent studies have found that Febprotects is effective against myocardial ischemia-reperfusion injury and has neuroprotective effects on cerebral ischemia-reperfusion in mice (17-19). Moreover, Feb ameliorates acute lung inflammation induced by lipopolysaccharide in rats and exerts anti-inflammatory action in KK-Ay Obese Diabetic Mice (20). Therefore, the present study is conducted to investigate the possible neuroprotective effect and cellular mechanism of Feb in a second injury after ICH. 

## Materials and Methods

***Animals***

Male C57BL/6 mice of 10 to 12 weeks were purchased from Chengdu Dossy Experimental Animals Co., LTD. (Chengdu, China). Animals were housed in a pathogen-free facility on a 12-hour light/dark cycle and provided *ad libitum* access to food and water. All experimental protocols for this study were approved by the Animal Ethics Committee of West China Hospital, Sichuan University (approval NO. 2020343A). After three days of adaptive feeding, the mice were randomly assigned to four groups. The Feb 50 group was given Feb (50 mg/kg) daily for five days, and the Feb 100 group was given Feb (100 mg/kg) daily for five days, while the ICH and sham groups were given an equal volume of saline. 

***ICH model***

The Feb tablets (Teijin Pharma Limited, Japan) were dissolved in saline and prepared into a solution of 5 mg/ml and 10 mg/ml. The ICH model was performed five days after administration of two dosages of Feb (50 mg/kg and 100 mg/kg) or saline. Mice were anesthetized with an intraperitoneal injection of pentobarbital sodium (45 mg/kg) and were then placed in a stereotactic frame (RWD Life Science Co, Shenzhen, China). A 1 mm hole was drilled 2.2 mm lateral to the midline and 0.3 mm anterior to bregma (21). A volume of 30 μl of autologous blood was collected from a tail. A needle was then inserted 3.3 mm into the right striatum through the hole. A volume of 5 μl of blood was injected into the target point at a rate of 2 μl/min with a microinfusion pump (WPI, Sarasota, FL, USA). The injection was then paused for 7 min before the remaining portion was delivered at the same rate. When the injection was completed, the needle was left in place for 10 min to prevent possible backflow. After the withdrawal of the needle, the skin was sutured. The mice in the sham group received all the above-mentioned procedures but the 30 μl saline was injected instead of fresh autologous blood (22). Body temperature was maintained at 37 ^°^C throughout all of these procedures.

***Brain tissue section***

Mice were sacrificed under deep pentobarbital sodium anesthesia. The brains were perfused with paraformaldehyde and soaked overnight. The brain was sliced into 1 mm thick slices using a sagittal brain section mold. 

***Behavioral testing***

Two behavioral tests, the neurobehavioral Evaluation Score and corner test, were performed at 72 hr post-ICH induction. Mice were examined using a previously published scoring system and monitored for appetite, activity, and neurological defects (details were shown in Table 1)(23). The device for the corner test is two baffles placed perpendicular to the plane, intersecting to form a 30^°^ angle. Then the mice to be tested were placed in the area in front of the baffle, and the plane was tapped to make the mice enter the included angle area. After entering the angle, the mice moved away from the angle in one direction by rotating their heads and torso. Under normal conditions, the mice were equally likely to turn to the left and the right. When one side of the nerve was impaired, the mice chose to turn to the opposite side. The probability of turning on one side was recorded to see if the mice had significant impairment of nerve function on one side(24). Each mouse repeated the experiment at least 10 times.

***Enzyme-linked immunosorbent assay***

Serum samples were collected 3 days following ICH or sham surgery. Tumor necrosis factor-α (TNF-α) and interleukin-1β (IL-1β) were measured in blood using a commercially available ELISA kit (Nanjing Jiancheng Bioengineering Institute, China). All samples and standards were assayed in duplicate according to the manufacturer’s instructions.

***Immunofluorescence staining***

The mice were deeply anesthetized and were transcardially perfused with 20 ml ice-cold PBS followed by 20 ml of 4% paraformaldehyde at 72 hr post-ICH. The whole brain was collected and then fixed in 4% paraformaldehyde for another 24  hr. Afterward, the brain was fixed in 20% sucrose solution until the tissue sank to the bottom followed by 30% sucrose solution for another 24  hr. After being frozen at -25 ^°^C, the brain was cut into 10-μm-thick coronal sections using a cryostat (CM1860; Leica Microsystems, Germany). To conduct double immunohistochemistry staining, the brain sections were incubated with primary antibody of anti-ionized calcium-binding adaptor molecule 1 (Iba-1, 1:100, Abcam), anti-nacht leucine-rich repeat protein 3 (NLRP3, 1:200, CST), anti-caspase-1(1:200, Invitrogen) overnight at 4 ^°^C. After being incubated with the appropriate secondary antibody (1:200, Bioss) at 37 ^°^C for 1 h, the sections were visualized and photographed with a fluorescence microscope (Olympus, Tokyo, Japan). 

***Fluoro-Jade B histochemistry***

FJB is a polyanionic fluorescein derivative that binds with high sensitivity and specificity to degenerating neurons. Briefly, sections were immersed in a graded series of alcohol solutions and distilled water, incubated in 0.06% KMnO_4 _for 10 min, rinsed in distilled water for 2 min, incubated in a 0.0004% solution of FJB (Chenmicon, Temecula, CA, USA) for 30 min, and observed under a fluorescence microscope (Olympus, Tokyo, Japan) at 450-490 nm. 

***TUNEL staining***

Terminal deoxynucleotidyl transferase-mediated dUTP-biotin nick end labeling (TUNEL) staining was performed on paraffin-embedded sections according to the manufacturer’s instructions. The total number of TUNEL-positive cells in the ipsilateral hemisphere was counted in three different fields for each section by an investigator who was blinded to the studies by light microscopy.

***Western blotting***

After mice were perfused with ice-cold PBS (0.1 M, pH 7.4) at 72 hr post-operation, the peri-hematoma tissues were collected and stored in a - 80 ^°^C freezer until use. Western blotting was performed as previously described. After sample preparation, equal amounts of protein were loaded onto an SDS-PAGE gel. After being electrophoresed and transferred to a PVDF membrane, the membrane was blocked for 2 hr at 37 ^°^C followed by incubation with the primary antibody overnight at 4 ^°^C. Then, the membrane was blocked with skim milk in PBS/T for 1 hr and incubated with primary antibody against anti-NLRP3 (1:1000, Abcam, MA, United States), anti-pro-caspase-1 (Asp210) (1:1000, Invitrogen, CA, United States), anti-cleaved-caspase-1((1:1000, Invitrogen, CA, United States) and anti-ASC (1:1000, Santa Cruz, CA, United States) and β-actin (1:2500, Santa Cruz, CA, United States). After blocking with 5% skim milk in PBS/T, the membrane was probed with the relevant antibody and visualized by enhanced chemiluminescence, according to the manufacturer’s instruction (Amersham, MA, USA). All data were analyzed using the software Image J. The images here have been cropped. See the supplementary materials for the original images.

***RNA extraction, library preparation, and sequencing***

As previously described, in brief, total RNA was extracted from the brain, and RNA-seq libraries were prepared according to the manufacturer’s kit. The library products corresponding to 200-500 bps were enriched, quantified, and finally sequenced on a NovaSeq 6000 sequencer (Illumina) with the PE150 model(25).

***Series test of cluster (STC) analysis***

The R package Mfuzz (version 2.58.0) was used to identify the genes between the Sham, Model, and Feb groups and perform STC analysis. STC analysis mainly aimed to study the trend characteristics of gene expression when the organisms changed in sequence or were stimulated by some external environment(26, 27).

***GO, KEGG, and Wiki pathways enrichment analyses***

To understand the enrichment of genes in significant STC cluster profiles, Gene Ontology (GO) annotation analysis, Wikipathways enrichment, and Kyoto Encyclopedia of Genes and Genomes (KEGG) pathway enrichment analyses of the achieved gene groups were performed by clusterProfiler (version 4.9.0.002) and GO.db (version 3.16.0). And the results were visualized by ggplot2 (version 3.4.2). 

***Statistical analysis***

For animal experiments, five to eight animals were randomly selected. All values were expressed as mean±SD. Prism 5 (GraphPad Software, La Jolla, USA) was used for statistical analysis. The two-tailed Student’s t-test was used for the comparison of two groups when the data were normally distributed and variances were equal. *P*<0.05 was considered statistically significant.

## Results

***Febuxostat treatment improved neurological outcomes in mice***

**Figure 1:**
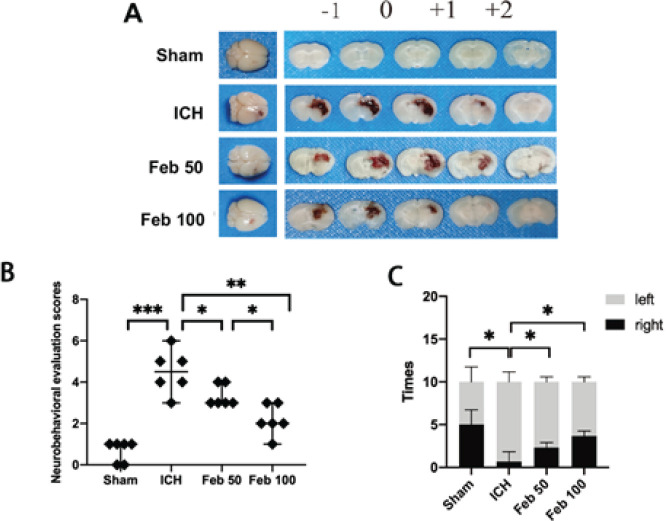
Febuxostat (Feb) improved neurocognitive impairment in mice after intracerebral hemorrhage (ICH) operation

Compared with the sham group, both the model group and the febuxostat group showed varying degrees of hemorrhage. And Feb improves hematoma resolution (Figure 1A). Neurological score was severely increased in ICH group compared with the sham group, which was impaired in Feb group (Figure 1B). Moreover, a significant increase in the percentage of right turns was found in ICH group, and was reduced in Feb group (Figure 1C). As described above, neurological functions were greatly improved by administration of Feb after ICH. Since the Feb 100 group showed better neurological outcomes, the dose was used in the following experiment.

***Trend expression analysis and enrichment analysis of genes***

**Figure 2:**
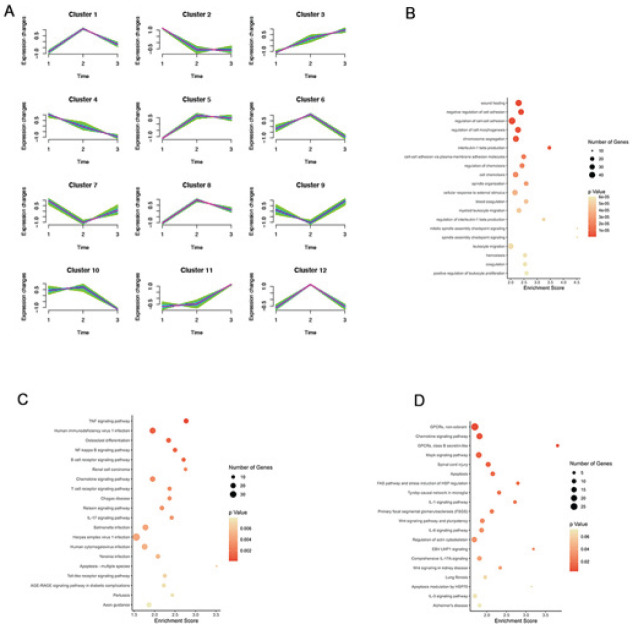
Bioinformatics analysis of febuxostat in cerebral hemorrhage in mice

As already reported in our previous study, to investigate the molecular mechanism of neuroprotection by Feb, gene expression profiling was performed (25). Here, we did specific trend expression analysis to investigate which genes changed significantly after Feb intervention. Each profile contained a cluster that had a similar changing tendency of genes. Twelve model profiles of genes were summarized (Figure 2A). 

Out of the 12 gene clusters, 5 clusters showed elevated gene expression after bleeding, and Feb intervention reduced the expression of these genes (clusters 1, 5, 6, 8, and 12). Two clusters showed decreased gene expression after bleeding, and Feb restored these gene expressions (clusters 7 and 9). We were more interested in which gene expression was reduced by Feb, so we chose cluster 12, in which the reduction was most pronounced, for analysis.

To predict the pathways of genes in Cluster 12, GO (Figure 2B), KEGG (Figure 2C), and WikiPathways (Figure 2D) analyses were performed. The top 20 most enriched pathways are shown in Figure 2 B-D. The KEGG pathway analysis revealed that genes were significantly enriched in the TNF signaling pathway, human immunodeficiency virus 1 infection, osteoclast differentiation, and NF-kappa B signaling pathway. Inflammation-related pathways are mostly enriched in GO and WikiPathways enrichment analyses (Figures 2C&D). As reported in previous studies (28), activation of neuroinflammation after cerebral hemorrhage is one of the main mechanisms leading to secondary injury. So next, our study focused on neuroinflammation to investigate the neuroprotective mechanism of Feb after cerebral hemorrhage.

***Febuxostat treatment reduced neuronal degeneration and neuronal apoptosis in brain tissues following ICH***

**Figure 3:**
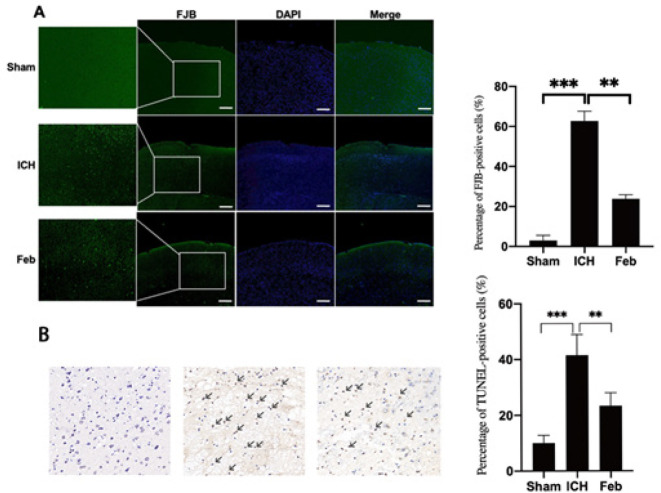
Febuxostat reduced neuronal degeneration and apoptosis in the *in vivo* model of intracerebral hemorrhage (ICH) of mice

The FJB assay was used to assess the protective capacity of Feb on neuronal cells. Few FJB-positive cells were observed in the sham group. However, FJB-positive cells were increased in the ICH group, which was attenuated by pretreatment of Feb (Figure 3A). Additionally, neuronal apoptosis was detected by TUNEL staining. The results indicated that the number of apoptotic neurons was higher than in the ICH group compared with the sham group, but was decreased in the presence of Feb (Figure 3B).

***Febuxostat treatment attenuated expression of inflammatory cytokines and inhibited activation of microglia and expression of NLRP3 in microglia in brain tissues following ICH***

**Figure 4:**
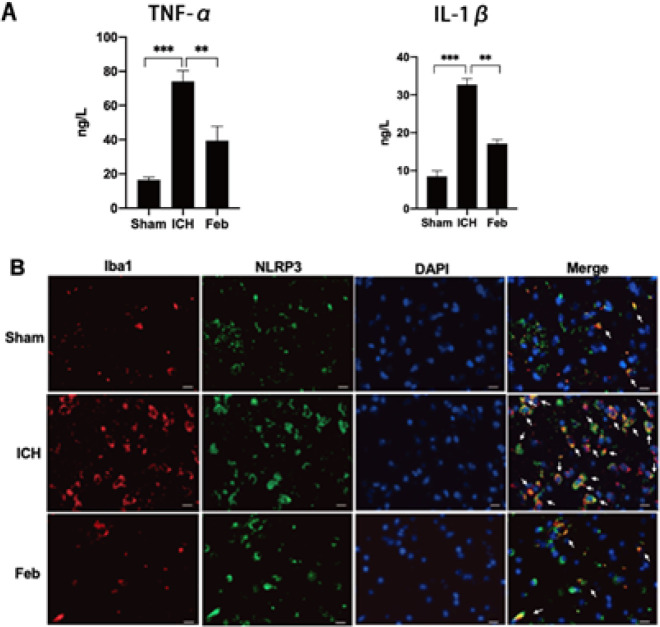
Febuxostat inhibited inflammatory cytokines levels and microglia activation after intracerebral hemorrhage (ICH) of mice

To define the role of Feb in neuroinflammation after ICH, we first detected the cytokine levels. The results showed that levels of TNF-α and IL-1β in brain tissues were significantly increased after ICH compared with the sham group. And Feb treatment attenuated both TNF-α and IL-1β levels (Figure 4A). 

A large number of endogenous activated microglia gathered in brain tissues after ICH, but Feb treatment significantly impaired the activation of microglia (Figure 4B). Additionally, double immunofluorescence staining showed increment of NLRP3 in microglia after ICH, which was reduced by Feb treatment (Figure 4B). 

***Febuxostat treatment inhibited activation of NLRP3/ASC/caspase-1***

**Figure 5:**
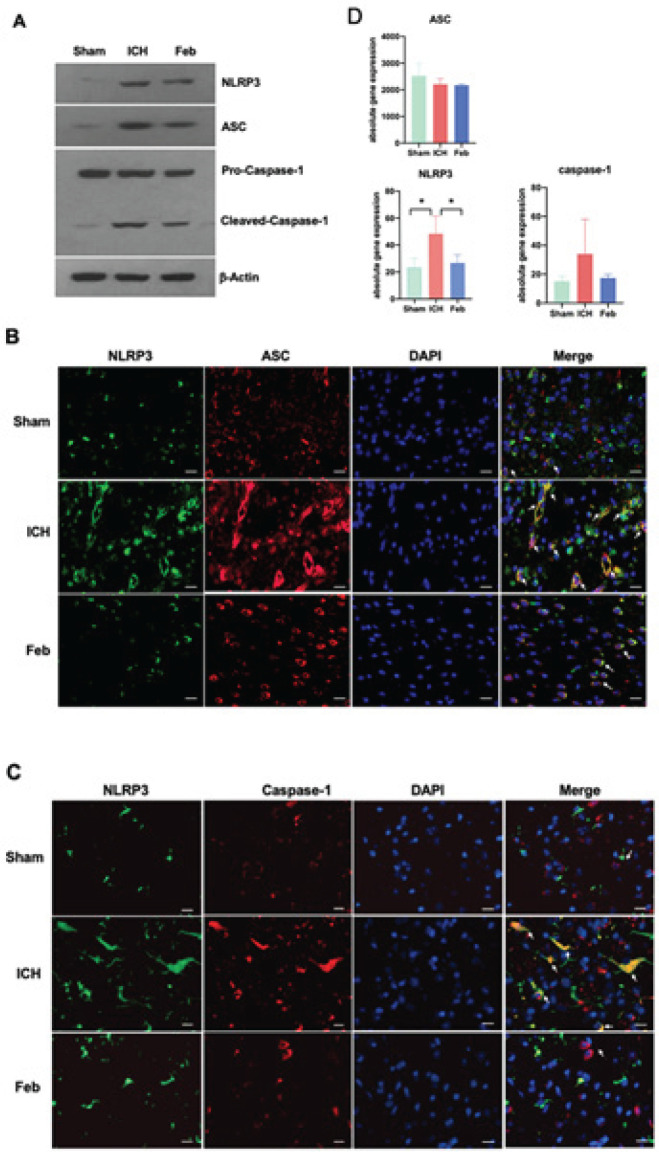
Febuxostat inhibited activation of NLRP3 inflammasome in the brain after intracerebral hemorrhage (ICH) of mice

To further explore the activation of the NLRP3/ASC/ caspase-1 pathway in brain tissues after ICH, we detected the protein samples from brain tissues around the hematoma by western blotting analysis. The expression of NLRP3 and ASC and the activation of caspase-1 (assessed by the appearance of cleaved-caspase-1) were increased after ICH, and were reduced by Feb treatment (Figure 5A). Consistently, double immunofluorescence staining showed that expression of NLRP3, ASC expressions, and caspase-1 were increased after ICH, and were reduced by Feb treatment (Figure 5B, C). Moreover, the results also revealed that the colocalizations of NLRP3, ASC, and caspase-1 were enhanced after ICH, but were attenuated by Feb treatment (Figure 5B, C). We also studied the expression of relevant genes at the transcriptional level. By analyzing the absolute quantification of the relevant genes in the transcriptome sequencing results, we found that at the gene level, NLRP3 and CASPASE-1 showed the same expression trend as at the protein level. However, the expression of ASC at the gene level was inconsistent with that at the protein level, suggesting that Feb may have interfered with ASC expression through other post-transcriptional mechanisms (Figure 5D). All the results suggested that Feb treatment inhibited activation of the NLRP3/ASC/caspase-1 pathway.

## Discussion

ICH accounts for approximately 10-20% of all strokes (29-32). Survivors suffer from severe and prolonged neurological dysfunction (33). ICH triggers a series of complex physiological and pathological events, including oxidative stress, inflammatory cell infiltration, cell necrosis, and apoptosis, which ultimately lead to brain damage, especially in the tissues around the hematoma (34). 

Feb is a xanthine oxidase inhibitor used clinically to lower uric acid (35). Due to its potent antioxidant activity, it has been found in some studies to have varying degrees of protective effects against myocardial ischemia-reperfusion (36), renal fibrosis (37), and renal brain ischemia-reperfusion (38). However, its protective mechanism in cerebral hemorrhage has been less studied. The present study clearly described that Feb ameliorated neurobehavioral disorders after ICH (Figure 1). The protective effect was dose-dependent, with a significant reduction in behavioral scores and bleeding area in the high-dose group (Figure 1A & B).

Trend analysis is a commonly used bioinformatics analysis method to analyze certain genes that show the same trend of change over time, with different drug interventions, or with changes in drug concentration (26). In turn, the function of these genes with the same expression trend is analyzed and predicted. By analyzing cluster 12, we found that the protection of Feb after cerebral hemorrhage is associated with many classical inflammatory pathways; for example, the TNF signaling pathway (Figure 2B). Tumor necrosis factor α (TNF-α) is a cytokine with pleiotropic effects on multiple cell types. It has been identified as a major regulator of the inflammatory response (39). In mice with cerebral hemorrhage, irisin ameliorated neuroinflammation and decreased TNF-α expression after cerebral hemorrhage (40). 

Moreover, FJB and TUNEL were performed to test neuronal degeneration and death in the brain. Both FJB-positive cells and TUNEL-positive cells were increased in brain tissues in the ICH group but were significantly aggravated by pretreatment of Feb (Figure 3A & B). These data indicated that Feb could protest against SBI after ICH.

A number of studies suggested that inflammation was closely related to the outcome and prognosis of ICH (41, 42). Feb has been shown to have anti-inflammatory activity in a variety of diseases (43, 44). It could reduce the degrees of glomerular injury as well as the IL-1β, IL-6, MCP-1, and ICAM-1 mRNA levels of KK-Ay mouse kidneys in diabetic kidney disease models (20). Moreover, administration of Feb could also attenuate ulcerative colitis by the inhibition of NF-κB, proinflammatory cytokines, and oxidative stress in mice (45). In the study, Feb treatment reduced inflammatory cytokines levels (TNF-α and IL-1β) in peripheral blood, suggesting Feb suppressed secretion of cytokines after ICH (Figure 4A). This is consistent with the results of KEGG enrichment analysis (Figure 2B). 

Different enrichment analyses have suggested that Feb is involved in the inflammatory pathway after brain hemorrhage. So we brought together our focus on microglia, the most important neuroimmune cells in the brain. Microglia were the primary immune defender of the CNS (3, 46), and participated in driving pro-inflammatory, oxidative, and cytotoxic stress, causing cell death and functional impairment (3, 4, 47). We found a number of endogenous activated microglia gathered in brain tissues surrounding the hematoma after ICH, however, pretreatment of Feb greatly impaired the activation of microglia (Figure 4B).

Inflammasomes are a group of large multiprotein complexes in response to the processing of pro-inflammatory cytokines and stimulate innate immunity (48). NLRP3 inflammasome is one of the best characterized cytosolic macromolecular complexes, composed of NLRP3, apoptosis-associated speck-like protein containing a caspase recruitment domain (ASC), and precursor caspase-1 (procaspase-1)(9). There are two processions for NLPR3 inflammasome activation. NLRP3 protein forms a complex with apoptosis-associated speck-like protein containing a CARD (ASC) and then binds to the cysteine protease caspase-1 to form the inflammasome (10, 49). In Alzheimer’s disease, the NLRP3 inflammasome assembles inside of microglia upon activation, leading to increased cleavage and activity of caspase-1, resulting in neurodegeneration and cognitive decline (50, 51). To further explore whether Feb modulates neuroinflammation via NLRP3 inflammasome, we performed double immunofluorescence staining and western blotting analysis. The double immunofluorescence staining showed Feb treatment significantly suppressed the increment of NLRP3 in microglia after ICH, which was reduced by Feb treatment (Figure 5). Moreover, Feb also reduced expression of ASC and NLRP3 in brain tissues after ICH (Figure 5B & C). Additionally, Feb could block the activation of caspase-1 and reduce the production of TNF-α and IL-1β (Figure 4A & Figure 5C). Furthermore, colocalization of NLRP3 versus ASC or caspase-1 was significantly reduced by Feb (Figure 5). Therefore, the study suggested that Feb protected against second brain injury after ICH probably through suppression of neuroinflammation via inhibiting NLRP3 inflammasome.

**Table 1:** The neurobehavioral evaluation of mice

Category	Behavior	Score
Appetite	Finished meal	0
	Left meal unfinished	1
	Scarcely ate	2
Activity	Walk and reach at least three corners of the cage	0
	Walk with some stimulations	1
	Almost always lying down	2
Deficits	No deficits	0
	Unstable walk	1
	Impossible to walk	2

A. Coronal sections of brain tissues after 72 hr post-ICH induction. B. Neurobehavioral evaluation scores of mice after 72 hr post-ICH induction. ***P<0.001, **P<0.01, n=6. C. The number of times the mice turned to the right in the corner test. *P<0.05, n=6. Feb 100, 100 mg/kg febuxostat. Feb 50, 50 mg/kg febuxostat. All data are expressed as mean±SD

A. Trend analysis of profiles. 1 represents the sham operation group, 2 represents the model group, and 3 represents the febuxostat group. B. GO bubble map demonstrates the top 20 pathways enrichment. C. KEGG bubble map demonstrates the top 20 pathways enrichment. D. Wiki pathways bubble map demonstrates the top 20 pathways enrichment

A. Fluro-Jade B (FJB) staining and percentage of FJB-positive cells. Scale bar: 100 μm. ***P<0.001 and **P<0.01, n=6. B. TUNEL staining and percentage of TUNEL-positive cells. Scale bar: 50 μm. Arrows indicate TUNEL-positive cells (dark brown) in the cerebral cortex. ***P<0.001, **P<0.01, n=6. All data are expressed as mean±SD

A. Levels of TNF-α and IL-1β in peripheral blood. ***P<0.001, **P<0.01, n=6. B. Double-immunofluorescence analysis was performed with antibodies for IBA1 (red) and NLRP3 (green), and nuclei were fluorescently labeled with DAPI (blue). Scale bar: 50 μm

A. Expression of NLRP3, ASC, and caspase-1 detected with indicated antibodies. B. Double-immunofluorescence analysis was performed with antibodies for ASC (red) and NLRP3 (green), and nuclei were fluorescently labeled with DAPI (blue). Scale bar: 50 μm. C. Double-immunofluorescence analysis was performed with antibodies for caspase-1 (red) and NLRP3 (green), and nuclei were fluorescently labeled with DAPI (blue). Scale bar: 50 μm. D. Quantification of the relevant genes in the transcriptome sequencing result. (*P<0.05, n=3)

## Conclusion

In the present study, we found that Feb improved neurocognitive function after cerebral hemorrhage in mice. By analyzing genes after hemorrhage, we found that Feb was more likely involved in neuroprotection after cerebral hemorrhage by affecting inflammation-related pathways. Our next study confirmed that Feb could reduce the level of inflammatory factors after cerebral hemorrhage and attenuate the activation of NLRP3 inflammasome, an important inflammatory molecule in neuroinflammation. Feb also reduced neuronal degeneration and neuronal apoptosis in brain tissues. In this study, we further revealed the protective effects of Feb after secondary injury in cerebral hemorrhage by bioinformatics and pharmacological methods.

## Authors’ Contributions

TJ Z and XTS M conceived and designed the experiments. Y B, Y Z, CY Z, ZW F, and Q W performed the experiments. Y B, HX S, Y Z, and CY Z analyzed the data and contributed to the manuscript draft. HX S and XH W drew the graphical abstract. B W and HX S critically revised the manuscript.

## Funding Sources

This work was supported by the Sichuan Province Science and Technology Program (2020YFH0059; 2019YFH0092), Peking Medical Award Fund (YXJL-2020-1159-0465), and Sichuan Provincial Hospital Association 2022 Young Pharmacist Research Special Fund Project (22010).

## Ethics Statement

All experimental protocols for this study were approved by the Animal Ethics Committee of West China Hospital, Sichuan University (approval NO. 2020343A).

## Conflicts of Interest

The authors declare they have no competing interests.
